# From definition to protection: dilemmas and reflections on the right to refuse treatment for patients with mental disorders in Chinese mainland

**DOI:** 10.3389/fpubh.2024.1410511

**Published:** 2024-08-08

**Authors:** Xiaofu Li, Xiaofan Li

**Affiliations:** ^1^School of Foreign Affairs and Law, East China of Political Science and Law, Shanghai, China; ^2^Department of Hematology, Fujian Medical University Union Hospital, Fuzhou, Fujian, China; ^3^INSERM U1160, Hopital Saint Louis, Université Paris Diderot, Sorbonne Paris Cité, Paris, France

**Keywords:** right to refuse treatment, patients with mental disorders, legal protection, medical practice, Chinese mainland

## Abstract

**Background:**

The case of “a multimillionaire who was sent to a psychiatric hospital after an argument with his son” has sparked heated debate in the Chinese mainland. This incident is particularly significant as 2023 marks the 10^th^ anniversary of the implementation of the Mental Health Law of the People's Republic of China. The focus of the ongoing debate, as brought to light by the aforementioned case, is centered on the right to refuse treatment for patients with mental disorders.

**Methods:**

This paper is a *post-hoc* study with a systematic analysis of literature and cases. To ascertain the relationship between the right to refuse treatment for patients with mental disorders and the Mental Health Law, the authors identified key information and data from both official government websites and reliable non-governmental information.

**Result:**

Both literature and practice have proven that the compulsory hospitalization rule under the Mental Health Law is a denial of the right to refuse treatment for patients who are compulsorily hospitalized. In the absence of changes to the law, compulsory hospitalization will inevitably lead to compulsory treatment in the Chinese mainland.

**Conclusion:**

According to the human dignity and self-determination right established in the Constitution of the People's Republic of China, patients who are compulsorily hospitalized have the right to refuse treatment. In the absence of a change in the law, given that no neutral review mechanism has been established for such patients and their treatment in the mainland, setting up an internal review mechanism is a more feasible way of protecting the right to refuse treatment for patients with mental disorders.

## 1 Introduction

In 2023, the death of multimillionaire Luo Wenzhong in Zhangjiajie City, Hunan Province, triggered heated debate within the Chinese mainland. Luo Wenzhong, 62 years old, was sent to a psychiatric hospital after a dispute with his son over family property. In the 2 months before his death, he had been complaining in various ways that he did not have a mental disorder and that it was the Second People's Hospital of Hunan Province that was biased, controlling his freedom and forcing him to take medication, which caused him serious injuries. His son, who sent him to the hospital for treatment, stated that he had commissioned a reputable organization to conduct an appraisal on his behalf. At present, the medical evaluation results are unknown. On August 25, 2023, Luo Wenzhong committed suicide while in the psychiatric hospital, which is a truly saddening tragedy (1).

In recent years, similar cases of controversy and even litigation arising from individuals being forcibly sent to psychiatric hospitals by family members or schools have become increasingly common in the Chinese mainland. Cases such as the one presented above not only expose procedural loopholes but also trigger reflection in the community on the restricted exercise of the right to refuse treatment for patients with mental disorders.

On October 10, 2010, the 16th World Mental Health Day, two non-governmental organizations, namely, Mental Illness and Social Observation and the Shenzhen Hengping Organization, released the Legal Analysis Report on China's Mental Illness Admission and Treatment System, which stated, “China's current mental illness admission and treatment system has huge flaws, and the situation is very confusing. The situation is very chaotic. This not only threatens public safety, but also puts everyone at risk of being admitted.” According to the report, the situation of “those who should be admitted are not admitted, and those who should not be admitted are admitted” leads to a waste of otherwise scarce medical resources and brings great pain and harm to the person concerned, intensifying social conflicts and leading to social disharmony (2).

On October 26, 2012, the 29th Meeting of the Standing Committee of the Eleventh National People's Congress of the People's Republic of China voted to adopt the Mental Health Law of the People's Republic of China, which came into effect on May 1, 2013. Article 30 of the Mental Health Law stipulates that “hospitalization for patients with mental disorders shall be on a voluntary basis.” This provision is believed to put an end to the incidence of “being psychiatrically ill” in the Chinese mainland (3).

The multimillionaire case is particularly significant as 2023 marks the 10th anniversary of the implementation of the Mental Health Law, and the case involves the right to refuse treatment for patients with mental disorders. This paper provides a comprehensive critique and recommendations on the exercise of the right to refuse treatment for patients with mental disorders in the mainland from the perspective of the psychiatric medical profession legislation and practice.

## 2 Materials and methods

### 2.1 Methods

This paper is a *post-hoc* study with a systematic analysis of literature and cases. To ascertain the relationship between the right to refuse treatment for patients with mental disorders and the Mental Health Law of the People's Republic of China, the authors decided to use certain academic tools and analyze judicial decisions. The authors identified key information and data from both official government websites and reliable non-governmental information.

### 2.2 Data collection

Data are collected from academic network platforms and official government websites.

Currently, laws do not clearly provide for the right to refuse treatment in the Chinese mainland. At the same time, the right to refuse treatment is rarely discussed in the academic discourse of the mainland. In October 2023, the authors searched the three major Chinese academic network platforms, namely, CHINA NATIONAL KNOWLEDGE INFRASTRUCTURE (https://www.cnki.net), WANFANG DATA (http://www.wanfangdata.com/), and CQVIP (http://www.cqvip.com), and found < 5 papers focusing on the analysis of the right to refuse treatment for patients with mental disorders.

Since January 1, 2014, each court in the Chinese mainland is mandated to upload the judgment of a case on their official website. The Provision of Publishing Judgment Documents on the Internet, which was released by the Supreme People's Court of the People's Republic of China, and its amendment (2016), mandated that judgments should be published online; for state secret, minor crime, mediation, or confirmation of the effectiveness of people's mediation agreement, divorce, or related minor child custody or guardianship, or commercial secret, the courts shall take some technical approaches to protect the privacy or secret. China Judgment Online (http://wenshu.court.gov.cn) is the official website for downloading judgments in the Chinese mainland. The authors selected 10 cases of people who were sent to psychiatric hospitals as one of the research bases for this study.

### 2.3 Data analysis

When checking the relevant data and information of the right to refuse treatment for patients with mental disorders, the authors pay attention to three psychiatric issues:

(1) What is the legislation purpose of the Mental Health Law of the People's Republic of China?

(2) Where are the grounds and boundaries of the right to refuse treatment for patients with mental disorders in the Chinese mainland?

(3) How can we better protect the right to refuse treatment for patients with mental disorders in the Chinese mainland?

## 3 Results

Literature and practice prove that the compulsory hospitalization rule under the Mental Health Law of the People's Republic of China denies the right to refuse treatment to patients who are compulsorily hospitalized. The Mental Health Law was not only introduced to define “patients with mental disorders” or “patients with severe mental disorders” but also to solve problems in the mental field by means of early social intervention. The right to refuse treatment for patients with mental disorders should be recognized and legally protected based on the respect for their autonomy and their right to health.

## 4 Discussion

### 4.1 Relevant regulations in the Chinese mainland and the problems they pose

As the core legislation in the psychiatric medical profession, the provisions of the Mental Health Law of the People's Republic of China relating to the hospitalization of patients with mental disorders are concentrated in articles 30, 31, and 83. Paragraph 1 of Article 30 of the Mental Health Law provides that “hospitalization for patients with mental disorders shall be on a voluntary basis.” Paragraph 2 of Article 30 provides for two situations where the medical institution or guardian has the power to “decide to take compulsory hospitalization for the patients with severe mental disorders”: first, “if an act that harms oneself has occurred, or there is a danger of harming oneself”; and second, “if an act that jeopardizes the safety of another person has occurred, or there is a danger of jeopardizing the safety of another person.” Moreover, Article 31 states that if a patient with mental disorder “has already committed an act of harming himself or herself or is in danger of harming himself or herself,” then the medical institution “shall hospitalize the patient as long as the guardian agrees.” Lastly, Article 83 specifically defines “patient with severe mental disorder” as “[a] patient with severe symptoms of the disease, resulting in serious impairment of the patient's social adaptation and other functions, inability to have a complete understanding of his or her own state of health or objective reality, or inability to handle their own affairs.” According to the Code of Practice for the Management and Treatment of Serious Mental Illnesses of the People's Republic of China (2012 Edition), serious mental illnesses “mainly include schizophrenia, bi-directional disorders, paranoid psychosis, schizoaffective disorders, etc.” The Code of Practice defines “serious mental illness” as “when the disease occurs, the patient loses awareness of the disease or control of behavior and may lead to behavior that endangers public safety and the personal safety of others. Long-term illness can cause serious damage to social functions.” The Notice of the Health Commission of the People's Republic of China on the Issuance of Work Specifications for the Management and Treatment of Severe Mental Disorders (2018 Edition) currently in effect in the Chinese mainland sets its own service recipients first as “diagnosed patients with six severe mental disorders, including schizophrenia, schizoaffective disorder, paranoid psychosis, bipolar (affective) disorder, mental disorder due to epilepsy, and intellectual disability with mental disorder.” Meanwhile, the official document holds that “patients who meet the second situation of Paragraph 2 of Article 30 of the Mental Health Law of the People's Republic of China and have been diagnosed and assessed as having a severe mental disorder by diagnosis and medical condition are not limited to the above six types.”

By combining the three articles and the two official psychiatry documents in the Chinese mainland, several conclusions can be drawn. First, the law divides the hospitalization of patients with mental disorders into two types, voluntary and involuntary, and defines “patient with severe mental disorder” as the main type of compulsory/involuntary hospitalization. Second, the Mental Health Law of the People's Republic of China makes “dangerousness” the core criterion for compulsory hospitalization. Third, although Article 83 of the Mental Health Law only defines “patients with mental disorders,” it does not specify the type or degree of severity. Although the Notice of the Health Commission of the People's Republic of China on the Issuance of Work Specifications for the Management and Treatment of Severe Mental Disorders (2018 Edition) lists the aforementioned six types of diagnosed patients with severe mental disorders, it does not specify or provide examples of the specific severity or degree of severity of these six types. Moreover, it allows patients “not limited to the above six types” to be considered as “patients with mental disorders.” In other words, the vagueness of the Mental Health Law and the Issuance of Work Specifications for the Management and Treatment of Severe Mental Disorders (2018 Edition) on “patients with severe mental disorders,” both of which are currently in force in the Chinese mainland, may expand the scope of the population that can be subjected to compulsory hospitalization.

The multimillionaire case in the background of this paper is representative of this situation, combining information from Luo Wenzhong and his son, the hospital, and other parties. Luo Wenzhong was admitted to the psychiatric hospital for two main reasons. One reason is that he was found to have a glioma and subsequently had a resection operation. According to medical judgment, an organic mental disorder of the brain may occur after surgery. The other reason is a video showing that he took a metal stick and smashed his son's computer, a TV, window glass, and other items, and there were blood stains on his hands. However, can these two reasons be enough evidence that Luo Wenzhong suffers from severe mental disorder? With regard to the first surgical factor, his sister and a number of other friends and relatives, including the community secretary, said that he had no abnormalities after surgery, denying the existence of sequelae. “It's possible” in the psychiatric medical profession does not mean it definitely exists. As for the violent vandalism video, it happened after Luo Wenzhong and his son had a fierce conflict. Between normal people, the phenomenon of vandalism because of family conflicts is not uncommon. Obviously, it would be too hasty to conclude that Luo Wenzhong suffered from a mental disorder or a severe mental disorder.

It is worth mentioning that the Mental Health Law adopts a model of compulsory hospitalization and compulsory treatment together. From the perspective of Chinese grammar, when the words “hospitalization” and “treatment” are put together, compulsory hospitalization implies compulsory treatment, and once a patient with mental disorder is unable to make a decision regarding hospitalization, the patient's right to refuse treatment is denied. In the psychiatric medical profession practice of the Chinese mainland, mental illness is often wrongly presumed to result in incapacitation, a presumption that completely denies the autonomy and informed consent of the patient with mental disorders and is thus deemed incapable of refusing treatment. The refusal of treatment by a patient with mental disorder is often regarded as a manifestation of their lack of disease cognition, rationality, and active legal capacity. Therefore, patients with mental disorders must accept treatment unconditionally regardless of whether they have the active legal capacity or ability to make decisions. Moreover, the psychiatric medical profession in the Chinese mainland holds a negative attitude toward refusal of treatment. On the one hand, refusal of treatment by a patient with mental disorder is believed to be a challenge to medical authority, which will weaken the dominance and control of physicians and hospitals over the treatment of illnesses and is not conducive to the maintenance of therapeutic order. On the other hand, from a medical point of view, refusal of treatment is not conducive to consolidating the effects of treatment and ensuring the continuity and stability of treatment and may result in the short- termination of treatment and a recurrence of the disease. These outcomes would make it difficult to achieve the dual purpose of compulsory treatment to safeguard public safety and the health of persons suffering from mental disorders (4).

Many types of mental illnesses exist, but not every mental illness has a corresponding drug and not many drugs are available to doctors in psychiatric clinics. With the developmental changes in society and the emergence of new types of patients with mental disorders, it is sometimes difficult for doctors to prescribe drugs corresponding to different diseases. For instance, drugs for schizophrenia may be inappropriately used for the treatment of diseases with similar symptoms. Indeed, antipsychotic drugs have generated great controversy since their inception. Medication can only eliminate or suppress psychotic symptoms, not necessarily cure mental illness completely, which is a typical “treating the symptoms but not the root cause (Chinese proverb)” approach. Despite the immediate therapeutic effect of many medications, discontinuing treatment increases the risk of a relapse of mental illness, and medication is relatively effective in managing acute psychiatric symptoms. For chronic mental illness, medication alone is less effective (5). Medications taken by patients with mental disorders may be completely ineffective and have significant side effects, thus posing greater risks to the physical and mental health of patients (6). In the case of post-traumatic stress disorder (PTSD), which has been a long-term concern for the authors (7), effective treatment includes both psychotherapy and medication, and the available evidence suggests that sedative-hypnotic drugs do not significantly improve insomnia symptoms in patients with PTSD (8). Meanwhile, research has shown that patients with mental disorders may not benefit from antipsychotic treatment, with some even deteriorating. Among all the controversies, the most prominent issue is the side effects of drugs: all antipsychotic drugs have a wide range of side effects. Compared with other drugs, antipsychotics often have more serious side effects, which often bring serious harm to the physical and mental health of patients with mental disorders; some of the adverse effects may even last for a lifetime without effective treatment, thus making the patients suffer immensely (9).

### 4.2 Origin and special significance of the right to refuse treatment

The authors have begun to reflect on the necessity of compulsory treatment and to legally challenge the authority of the government and medical institutions to impose treatment, armed with the right to refuse treatment. Accordingly, do patients with mental disorders who are compulsorily hospitalized enjoy the right to refuse treatment in the course of treatment? If patients with mental disorders enjoy the right to refuse treatment, what is the source and special significance of having such right? At the same time, what are the limitations of this right?

Outside the Chinese mainland, refusal of treatment as a right of a patient with mental disorder is recognized in many national and regional legislations. Paragraph 4 of Principle 11 of the United Nations Principles for the Protection of Persons with Mental Illness and the Improvement of Mental Health Care explicitly states that “a patient has the right to refuse or stop treatment.” Although it does not, for the time being, have the legal force of an international treaty, as the most complete international standard for the protection of the rights of persons suffering from mental disorders, the document serves as a guide to national legislation and mental health care practice.

In the United States, the right of hospitalized psychiatric patients to refuse treatment was once considered by scholars to be the most important issue in the psychiatric medical profession (10). However, this right has also been the subject of considerable controversy and disagreement between the medical and legal professions (11). Since the 1970s, the right to refuse treatment has gradually been recognized by domestic courts in the United States, and state legislation has generally recognized this right, with strict substantive and procedural protections (12).

Theoretically, the right to refuse treatment can be protected through the principle of informed consent; specifically, legal remedies are available through tort rules on the grounds that the treatment has not been consented to by patients with mental disorders. However, the traditional view in the Chinese mainland is these hospitalized patients with mental disorders are incapable of rationally making treatment decisions because of their mental problems and are thus not protected by the principle of informed consent (13). In fact, refusal of treatment is a proper part of the right to informed consent and medical autonomy, and there is no need to overemphasize the right to informed consent in situations where it can be fully respected and protected. However, in the context of compulsory treatment, especially under current legal provisions in the Chinese mainland, the right to medical autonomy for patients with mental disorders is excluded, and the ethical and legal rules of informed consent are no longer applicable: the treatment does not require the consent of patients with mental disorders. The authors argue that fundamental rights in the Constitution of the People's Republic of China and other laws can serve as a basis for exercising the right to refuse treatment.

The right to refuse treatment derives first and foremost from the constitutional right to human dignity and self-determination. This right cannot be denied simply because a person is suffering from a mental disorder; even patients who are compulsory hospitalized should have their right to refuse treatment recognized under certain conditions. Article 38 of the Constitution of the People's Republic of China states: “The human dignity of citizens of the People's Republic of China is inviolable. It is forbidden to insult, defame and falsely accuse citizens by any means.” This is the most important provision on human dignity in China's legal system. From a grammatical point of view, the first sentence of Article 38 can be interpreted to mean that China has made the protection of human dignity a norm, while the second sentence is in the form of a prohibition, which is a complementary explanation of how to protect human dignity. One manifestation of the protection of human dignity in society is its transformation into rights and freedoms, or what most Chinese scholars consider to be personality rights (14). Taking an overview of the mainstream theories of Chinese and foreign jurisprudence, the right of personality, as a historical and generalized right, has two main types: broad and narrow senses. In the broad sense, the right of personality is regarded as broadly encompassing the rights to life, body, chastity, honor, credit, name, and portrait, as well as rights closely related to private life, such as the right to privacy. In the narrow sense, the right of personality has different contents under different norms in different countries but generally include the rights to name, reputation, honor, and portrait, as well as the right to privacy, the right to self-determination, and other rights that are closely related to personality values (15). The right to self-determination is also a concept in Chinese laws. It derives from the dignity of the individual and the freedom of will that they enjoy and is the right of the individual to decide on matters within their own sphere without interference from others. Based on the right to self-determination, it inevitably gives rise to the right to take the initiative in forming one's own lifestyle and the right to passively counteract unlawful interference by the state. The result is that individuals are allowed to decide for themselves and be responsible for their own opinions and actions. Thus, the right to self-determination necessarily includes the exclusion of undue interference by public power in the freedom and will of the individual, including compulsory treatment (16).

In the academic world of the Chinese mainland, scholars in different fields have carried out more in-depth research on the issue of patients' capacity to give informed consent and its judgment standard. Some scholars believe that the patient's consent is a prerequisite for effective medical treatment, and the patient's ability to consent is a necessary condition for the validity of consent (17). Some scholars, from the perspective of comparative law and in accordance with the general principles of some developed countries, believe that it is inappropriate to determine the patient's ability to give consent on the basis of civil capacity and that it should be based on whether or not the patient has the ability to comprehend the content, significance, and effect of the consent (18). Some scholars also believe that interventional medical activities involve the disposition of their own rights; the theory does not need to have the ability to act, as long as the patient knows the risk of the relevant diagnosis and treatment behavior, the consequences of the necessary discernment, and understanding of the ability (19). Scholars who advocate for the protection of the rights of minors argue that the medical field usually presumes that minors lack the capacity to make medical decisions, which restricts their rights, especially the minors have the ability to truly understand, judge, and decide on their illness and medical treatment (20).

The essence of the independent theory of patient's capacity for informed consent is to distinguish the concept of such capacity from the active legal capacity, mental capacity, and identification capacity in tort law as well as form the theory of the patient's informed consent capacity that is specifically applicable to the medical field. The aim is to provide theoretical support for the concretization and specialization of the basis for determining the capacity for informed consent.

At the jurisprudence level, the capacity for informed consent independently is favorable to the determination of the validity of medical (treatment) decisions. In the Chinese mainland, the active legal capacity probably reflects the status of mental capacity, that is, the existence of the active legal capacity is for the sake of the simplicity of practical operation, which roughly corresponds to the reality of the civil subject's mental capacity. However, an increasing number of scholars believe that mental capacity and active legal capacity cannot be equated; that is, whether or not a person possesses full capacity for civil behavior, their mental capacity may not necessarily correspond to their active legal capacity (21). In the medical field, the capacity for informed consent should not be considered simply for the sake of operational simplicity but considered and evaluated on the basis of individualized judgment and respect for individual autonomy.

First, as far as the effectiveness of behavior is concerned, whether one has the capacity for informed consent determines whether the patient's consent to medical treatment is valid. In the field of legal acts in general, the standard for determining capacity is followed, but there are also cases in which a person with full capacity lacks the capacity to act in a particular situation. Even if a permissible act is performed by a normal person, it is not always valid. As a rule, the patient must first be informed of the necessity and danger of the infringement in question. Therefore, even if the patient does not have full capacity, the permissible act should be valid as long as they have the appropriate cognitive capacity. Therefore, in the medical field, enough attention and careful treatment must be paid to assessing the capacity for informed consent. It should involve flexible identification rather than a rigid application of the standard of active legal capacity. Second, in terms of the subject's interests, the capacity for informed consent should not be limited to the boundaries of age, intelligence, or mental state. Even if the person does not have fully legal capacity, it is possible to recognize capacity for individual cases, which can make the medical decision of the person valid and more effective in protecting their interests. In other words, even minors, or people under guardianship, may still have the ability to consent to medical matters. Just as a person with fully legal capacity may not have the capacity to consent to a particular act under certain circumstances, a person with a psychiatric disorder may have the capacity to consent to a particular act under certain circumstances; in fact, this is the normative expression of the relationship between active legal capacity and informed consent capacity (22). In conjunction with cases in the field of German civil law, “if, according to a minor's mental and moral maturity, he or she is able to gauge the aggression as well as the significance and consequences of consenting to the aggression, it is sufficient for the minor to express his or her permission.” However, such consent must be validly premised on an explanation of the necessity, risk, and possible harm of the medical act (23).

At the value level, informed consent capacity is independently said to be beneficial to the protection of patients' free will. Compared with active legal capacity, informed consent capacity can more deeply manifest the core value of medical law—respect for the patients' free will—which focuses on the protection of their rights and interest. In the traditional system of assessing active legal capacity, especially concerning the security of the transaction and consideration of the counterparty's interests, scholars have pointed out that it tends to be overly restrictive. Since the necessity of the active legal capacity in the civil system has been questioned, in the medical field, when assessing a patient's capacity for informed consent, it is inappropriate to rigidly and directly apply the concept of active legal capacity. First, the independence of informed consent capacity is more in line with the requirement that the “commitment to prevent the illegality of medical behavior” must be based on personal consent. Commitment behavior in the medical field is a specific embodiment of informed consent, but the patient's commitment is to medical behavior rather than medical results. Once the patient has given consent to the medical decision, because such consent has the legal legitimacy of medical behavior, it eliminates the possible existence of an illegal act. However, the promise refers to the medical treatment itself, not to its outcome (24). Second, the independence of informed consent is more conducive to the willingness to make autonomous decisions. Considering the patient's needs in terms of their immediate interests, and thus protecting their best interests, is the basis of the value of the right to informed consent. Every person has the right to make autonomous decisions about their own body and to accept or refuse medical treatment. This right belongs exclusively to the patient, and no one (including family members and physicians) may force medical treatment on a patient against their will (25). Returning to the patient's own will to make a substantial determination and obtaining their cooperation, thus enabling better completion of medical matters, is more in line with the patient's actual needs or best interests. At the same time, it also helps prevent legal representatives from abusing their power of consent by not making decisions or choices based on the agent's values, which, even in the so-called best interest, may be contrary to the patient's own wishes and lead to other unnecessary disputes. There is no doubt about the personal and significant nature of medical matters for the patients themselves, so their will and choice of whether to accept them should be respected and protected as a matter of course.

### 4.3 Discussion of dangerousness and assessment of patient ability

The essence of the right to refuse treatment is respect for the will and choice of the patient. Therefore, patients who are voluntarily hospitalized certainly enjoy the right to full informed consent, including the right to decide to accept or refuse specific treatment measures. The right to refuse treatment should not be denied to patients who are compulsory hospitalized but should be recognized within certain limits.

Although the Mental Health Law of the People's Republic of China considers dangerousness as the core criterion for compulsory hospitalization, the authors argue that dangerousness should not be used as the only criterion for patients with mental disorders. In Article 30 of the Mental Health Law, “harming oneself” and “jeopardizing the safety of others” should be interpreted as life-threatening or serious bodily harm, and other minor physical harm or emotional harm should be excluded. Article 30 likewise refers to dangerousness as having both high likelihood and urgency, which is understood as imminent danger. Typical examples are suicide or violent assault. To determine whether a patient with mental disorder is dangerous, certain scholars argue that such assessment can be carried out from five aspects: first, the type of behavior (including physical assault, behavioral threats, verbal threats, property damage, and purposeless harm), second, the frequency of the behavior; third, the recency of the behavior (the period of time prior to the detection of the disorder); fourth, the manner of harm (including the type of violence used); and fifth, the target of the behavior (the person himself or others, or both) (26). Some foreign scholars, such as Appelbaum, have added the person's own factors to the consideration of dangerousness, including the patient's impairment of active legal capacity (27).

Although there has been extensive discussion on the criterion of dangerousness, it would seem that there is nothing wrong with the compulsory medical treatment of persons with mental disorders who may cause harm to themselves or others. However, is this really the case? Is the application of the dangerousness principle to the compulsory treatment of persons with mental disorders discriminatory?

Assume that there are two persons, A and B, both having violent tendencies. A is not mentally impaired and is incarcerated to serve his sentence. Although A is still a danger to others, he will be released at the end of his sentence. B has a severe mental disorder (assumed to be bipolar disorder) and is admitted to a closed psychiatric hospital. B will remain in the hospital for compulsory treatment as long as he is considered a danger to others. What exactly is the justification for A's freedom after serving his sentence while B, who suffers from a mental disorder, is incarcerated indefinitely under mandatory confinement? If the only justification for imprisoning B is that he is still a danger to others, then A should also remain incarcerated. However, it is against the law to continue to imprison A after he has served his sentence. It might be argued that the frequency and predictability of B's violent behavior makes patients with mental disorders a population subject to special treatment. However, is the risk of violent behavior only high among patients with mental disorders? Most violent behavior is actually committed by people without mental disorders, and there is insufficient evidence to prove that violent behavior is any more predictable in patients with mental disorders than it is in people without mental disorders, for example, domestic violence and alcoholism. Even if violent behavior is proven in patients with mental disorders, why should they be forced to take precautions when people with domestic violence and alcoholism are not forced to take precautions? The crime sanctioning system in democratic countries provides a good example, but realized in a completely opposite direction: those accused of violent crimes are considered innocent until proven guilty by courts. Few laws allow for the imprisonment of innocent people, even if they are recognized as a risk of harm to others in the future (28).

Based on the comprehensive research results in the Chinese mainland, the definition of a patient's informed consent capacity is mostly linked to the civil law of active legal capacity and mental capacity. In view of the special nature of medical risks, some Chinese scholars believe that it is necessary to distinguish the patient's informed consent capacity from the general sense of active legal capacity or mental capacity so as to provide theoretical support for determining the patient's informed consent capacity in medical practice. In essence, the independent judgment of a patient's capacity for informed consent has two meanings: the first is to respect the patient's autonomy and their right to provide consent, and the second is to minimize medical risks. From the perspective of ensuring the legality of medical procedures, it involves effectively informing patients of medical information and treatment programs. Through informed consent, the patients have the capacity to choose, decide, and bear the consequences of their decisions. For both doctors and patients, the proper understanding and application of informed consent criteria can contribute greatly to enhancing the doctor–patient relationship and thus achieve a more harmonious, mutual trust, and win-win situation. For some specific civil subjects, although they may lack full capacity due to factors such as age, intelligence, mental state, or physical exhaustion, their judgment may not be so full. Nonetheless, it is important to recognize and respect their remaining capacity to make decisions. In the content of medical care, their corresponding rights should also be given full attention and protection. For example, for adult patients, when there is an abnormal or unreasonable reaction, the initial judgment of their ability to give informed consent needs to be specifically communicated and to rely on the clinical judgment of doctors. For underage patients, based on the efficiency and fairness of the legal value of the foothold, if it is related to an important matter or facing a significant risk of death, then strict standards of active legal capacity must be adopted; if facing a lower risk, then a more lenient standard of active legal capacity will be allowed, that is, different standards of capacity are applied to different types of medical acts (29).

In the psychiatric medical profession, patients with mental disorders should be assisted in normalizing their lives by applying evaluation mechanisms appropriate to their capacity. In the area of legal conduct, some Chinese scholars have already proposed that psychiatric persons with disabilities should no longer be characterized as persons without legal capacity. Protecting the rights and interests of adults with mental disorders through a system of restricted legal capacity rather than incapacity not only eliminates the problem of over-restriction inherent in the system of incapacity, but it also greatly facilitates their ability to live a normal life immediately and freely as their mental or meaning capacity is gradually restored (30).

First, in terms of philosophy, based on the value of ensuring the normalization of the social life of patients with mental disorders and their self-determination, they should be guaranteed the same capacity for self-determination as normal people so they can have a sense of undifferentiated social integration. This is not only the embodiment of the basic spirit of safeguarding human rights but also a requirement of the tangential nature of medical behavior. Second, in terms of pathways, the specific assessment and identification of the informed consent capacity of patients with mental disorders relies, to a great extent, on the proactive intervention and patient questioning of doctors in the course of medical treatment, which enables them to gain an in-depth understanding of the patient's underlying preferences, values, and core beliefs (31). The United Nations Convention on the Rights of Persons with Disabilities changes the legal presumption of incapacity attached to the label “disability” and requires all persons with disabilities to enjoy the presumption of competence and legal capacity as a matter of human rights law, establishing the principle of “equality for all.” It also requires member states to comprehensively replace the substitute decision-making model with the assisted decision-making model and respect the wishes and preferences of persons with disabilities to the greatest extent.

### 4.4 Protection mode of the right to refuse treatment in the Chinese mainland

The key to protecting the right to refuse treatment lies in clarifying both the content of the right and, more importantly, the conditions and procedures so as to prevent the right from being excessively restricted. From extraterritorial experience, including that of the United States, the protection of the right to refuse treatment is mainly based on both substantive and procedural aspects. The substantive aspects specify the conditions and circumstances for restricting the right to refuse treatment, and the procedural aspects stipulate the minimum due process that should be followed in restricting the right to refuse treatment.

From the perspective of extraterritorial experience, the path of legal regulation of compulsory treatment can be divided into three. The first mode is the model of separating compulsory hospitalization and compulsory treatment, whereby the compulsory treatment of hospitalized patients must be subject to independent review. This model is adopted by some common law countries. The second mode recognizes the right to refuse treatment for patients with mental disorders who are forcibly hospitalized, and the exclusion of the patients' right to refuse treatment (by means of compulsory treatment) should comply with both substantive rules and procedural requirements. For example, most Canadian provinces and some European countries recognize the right to refuse treatment in practice to varying degrees, for example, the Public Health Code in France. Third, psychiatric medication and other specialized treatments are specifically regulated, for example, the Mental Health Act 1983 (as amended in 2007) in England and Wales. In practice, it is precisely because of the abuse of antipsychotic drugs and their side effects, which cause patients with mental disorders to suffer serious harm to their health, psyche, and human dignity, that many of them have begun to seek legal remedies in an attempt to terminate or refuse medication they do not want to undergo. The right to refuse treatment has begun to gain traction as a legal weapon in the fight against coercive treatment and is gradually gaining ground in legislation and judicial practice.

In the Chinese mainland, the compulsory hospitalization and treatment of patients with mental disorders is based on a combination model, whereby compulsory hospitalization implies compulsory treatment and the patient's compulsory treatment is not subject to an independent process of assessment or review. While this model has the advantages of simple procedures, low costs, and ease of treatment, its drawbacks should not be overlooked, such as neglecting the relative independence of hospitalization and treatment and, in particular, neglecting the fact that the patient who is forcibly admitted to the hospital may still have the ability to decide on their treatment. However, it is undeniable that the combined model is more in line with the current situation of mental healthcare services in the Chinese mainland and can avoid the disadvantages of the separate model, such as the high costs and tedious procedures. At the same time, the Mental Health Law of the People's Republic of China does not regulate medication and electro-convulsive therapy in any way, effectively granting medical institutions and psychiatrists broad and unfettered discretionary power over treatment. In the mainland, the compulsory treatment of patients with mental disorders is hardly regulated by law. Against this background, it is particularly important to recognize the right of patients with mental disorders to refuse treatment and to strengthen the protection of that right. On the one hand, through the right to refuse treatment, the right to confront and reverse constraints on compulsory treatment is realized; on the other hand, through the recognition of the right to refuse treatment and the protection of its procedures, the procedural constraints on compulsory treatment are indirectly realized.

Specifically, the restriction of the right to refuse treatment shall be limited to cases where the mental illness of the patient with a mental disorder constitutes a danger to their own interests, the interests of others, or the public interest. In such cases, the right to refuse treatment may be excluded from the exercise of the right to compulsory treatment of patients with mental disorders in order to protect their own interests, the interests of others, and the public interest. In this regard, United Nations Principles for the Protection of Persons with Mental Illness and the Improvement of Mental Health Care stipulate that “a proposed plan of treatment may be given to a patient without a patient's informed consent if the following conditions are satisfied: (a) The patient is, at the relevant time, held as an involuntary patient; (b) An independent authority, having in its possession all relevant information, including the information specified in paragraph 2 above, is satisfied that, at the relevant time, the patient lacks the capacity to give or withhold informed consent to the proposed plan of treatment or, if domestic legislation so provides, that, having regard to the patient's own safety or the safety of others, the patient unreasonably withholds such consent; and (c) The independent authority is satisfied that the proposed plan of treatment is in the best interest of the patient's health needs.” Clearly, the United Nations Principles for the Protection of Persons with Mental Illness and the Improvement of Mental Health Care does not consider compulsory hospitalization to be a means of coercive treatment. On the contrary, certain conditions must be met in order to exclude the patient's right to informed consent or the right to refuse treatment because the Mental Health Law of the People's Republic of China uses dangerousness as the main condition for compulsory treatment. However, if the condition of a mentally disordered patient has been alleviated or effectively controlled by treatment and is no longer dangerous, then there is no justification for continuing to take compulsory treatment and thus denying the patient's exercise of their right to refuse treatment. Therefore, even for patients with mental disorders who are compulsory hospitalized, if the conditions for continued compulsory treatment are not met, then the patient's exercise of the right to refuse treatment should not be denied.

At the same time, the Chinese law should provide minimum procedural protections for the exercise of refusal of treatment by patients with mental disorders. From the experience of the United States, although states have provided strict judicial procedural protections for the restriction of the right to refuse treatment, a number of jurisprudences recognize that internal administrative hearings and even restrictions on the refusal of treatment based on the professional judgment of a doctor meet the requirements of due process. As far as the Chinese mainland is concerned, its Mental Health Law stipulates that the compulsory hospitalization of patients with mental disorders does not need to be examined and decided by a court or other neutral body (including courts and independent administrative bodies). In addition, the guardian or medical institution enjoys the right to decide on compulsory hospitalization, and the medical institution and the doctor enjoy the full decision-making power as to whether or not to treat the patient after their hospitalization as well as what kinds of therapeutic measures to take. In other words, the compulsory treatment of patients with mental disorders after hospitalization takes the form of a professional judgment model, in which the doctor can affirm or deny the patient's refusal of treatment based on their professional judgment.

Taking into account the serious side effects that compulsory treatment may have on patients with mental disorders, as well as the protection of patients' rights, more adequate procedural safeguards should be provided for the exercise of patients' right to refuse treatment. As far as the legislation of the Chinese mainland is concerned, the protection of the right to refuse treatment cannot be legislated to introduce a judicial review model. In the absence of changes in the legislation, a feasible approach would be to adopt an internal review model, thereby providing a minimum of procedural protection for patients with mental disorders. Specifically, medical institutions should set up a relatively independent department, for example, a psychiatric healthcare review board, whose members may be experts from various fields, such as medicine, law, ethics, social work, and so on. These experts can help review the applications or claims of patients with mental disorders who refuse to be treated in order to decide whether or not to continue to subject the patients to compulsory treatment. The review by the psychiatric healthcare review board is conducted in a meeting format (not limited to offline), and the review process shall focus on the following issues: (1) whether the patient's condition is in remission, (2) whether the patient is dangerous, (3) the necessity and effectiveness of continuing the treatment, (4) the side effects of the treatment and their severity, and (5) whether the patient has the capacity to refuse the treatment. In addition to the written review, the psychiatric healthcare review board may hear the opinions of the attending physician, the patient, or the patient's close relatives and make a decision on whether to agree with the patient's refusal of treatment by a majority vote based on a full consideration of the specific circumstances of the case. At the same time, patients have the right to file a lawsuit in court against a medical institution that denies the refusal of treatment to a patient with a mental disorder, thereby providing a judicial remedy for the exercise of the right to refuse treatment.

## 5 Conclusion

According to the right to human dignity and self-determination established in the Constitution of the People's Republic of China, patients who are compulsory hospitalized should enjoy the right to refuse treatment. In the absence of change in the law, compulsory hospitalization will inevitably lead to compulsory treatment, and given that no neutral review mechanism has been established for both compulsory hospitalization and treatment in the Chinese mainland, it is impossible to introduce an extraterritorial model of judicial review into the protection of the right to refuse treatment. A more feasible approach would be to set up an internal review mechanism as a means of protecting the right to compulsory medical treatment of patients with mental disorders.

## Data availability statement

The original contributions presented in the study are included in the article/supplementary material, further inquiries can be directed to the corresponding author.

## Ethics statement

Ethical review and approval was not required for the study on human participants in accordance with the local legislation and institutional requirements. Written informed consent from the patients/participants or patients/participants' legal guardian/next of kin was not required to participate in this study in accordance with the national legislation and the institutional requirements.

## Author contributions

XiaofuL: Conceptualization, Data curation, Methodology, Writing – original draft. XiaofaL: Formal analysis, Writing – review & editing.
